# Effect of the Ti_2_CT_*x*_ (T_*x*_ = O, OH, and H) Functionalization on the Formation of (TiO_2_)_5_/Ti_2_CT_*x*_ Composites

**DOI:** 10.1021/acs.jpcc.4c06909

**Published:** 2024-12-19

**Authors:** Néstor García-Romeral, Ángel Morales-García, Francesc Viñes

**Affiliations:** Departament de Ciència de Materials i Química Física & Institut de Química Teòrica i Computacional (IQTCUB), Universitat de Barcelona, c/Martí i Franquès 1-11, 08028 Barcelona, Spain

## Abstract

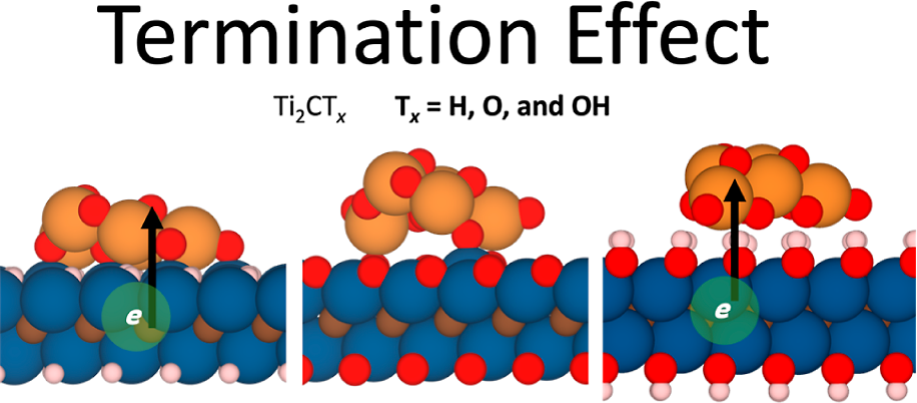

First-principles density functional theory calculations are carried out on the (TiO_2_)_5_ cluster supported on the Ti_2_CT_*x*_(0001) surface with different chemical terminations, *i.e.*, −H, −O, and −OH, to study the interaction and understand the Ti_2_CT_*x*_ functionalization effect on the formation of (TiO_2_)_5_/Ti_2_CT_*x*_ composites. Results show an exothermic interaction for all cases, whose strength is driven by the surface termination, promoting weaker bonds when the MXene is functionalized with H atoms. For Ti_2_CH_2_ and Ti_2_C(OH)_2_ MXenes, the interaction is accompanied by a charge transfer towards the titania cluster. All adsorptions are accompanied by a significant structural deformation of the titania nanocluster. The analysis of the density of states of (TiO_2_)_5_/Ti_2_CH_2_ and (TiO_2_)_5_/Ti_2_C(OH)_2_ composites shows a clear almost metallic character with titania-related states close to the Fermi level. However, for (TiO_2_)_5_/Ti_2_CO_2_, the band positions are similar to those of a Type-I heterojunction. Overall, the MXene surface termination influence on the TiO_2_/MXene interaction is unveiled, providing more stable composite formations when the MXene surface is functionalized with −H and −OH groups, where the adsorption process is accompanied by significant charge transfer.

## 1 Introduction

In 2011, a new family of low-dimensional transition-metal carbides, nitrides, and carbonitrides materials, known as MXenes, was discovered.^1,2^ These materials can be synthesized by the selective chemical etching of A element from MAX phases, with the M_*n*+1_AX_*n*_ chemical formula, where M stands for an early transition metal, A corresponds to elements from groups XIII or XIV, X is C and/or N, and *n* ranges from 1 to 4, determining the MXene thickness.^2−4^ After disassembling the MAX phase, MXene layers are formed having the M_*n*+1_X_*n*_T_*x*_ formula, where T_*x*_ denotes chemical groups (or terminations) bonded on the MXene (0001) surfaces as a result of the synthesis conditions, commonly being −F, −H, −O, and −OH.^5,6^ It is worth highlighting that some recently published works have unveiled the presence of O atoms within the X MXene layers.^7−9^ Additional treatments can be applied to efficiently remove T_*x*_,^10,11^ resulting in pristine MXene surfaces, M_*n*+1_X_*n*_.^5,10^ The great expectation generated by MXenes in the scientific community is due to their broad versatility and range of applicability. MXenes exhibit a wide range of properties dependent on their composition, thickness, and surface terminations.^2^ Among the different applications of MXenes, some remarkable ones are their use as electromagnetic interference (EMI) shielding materials,^12−14^ in CO_2_ abatement,^15,16^ in water purification,^17^ their use in alkali-ion batteries,^11,18−20^ for lubrication,^21,22^ as gas- and biosensors,^23,24^ and in thermo-, electro-, and photocatalysis.^25−29^

MXenes’ stability gets normally compromised when exposed in aqueous environments since the oxidation of Ti-based MXene surfaces leads to structural degradation and loss of performance on their applications, creating TiO_2_/Ti-based MXene heterostructures.^30,31^ On the other hand, the partially oxidized Ti-based MXenes have proven to open the range of applications as photocatalysts in CO_2_ photoreduction,^32−34^ H_2_ generation,^35−38^ and N_2_ fixation.^39,40^ It is worth highlighting that this oxidation process can be controlled to obtain interfaces of TiO_2_/Ti-based MXene with different TiO_2_ polymorphs and surfaces.^41^ Indeed, it has been reported that these photocatalytic applications are rooted in the metal–semiconductor junction formed at TiO_2_/MXene interfaces. This junction enhances an adequate separation of electrons and holes while maintaining the redox ability of charge carriers in an efficient way.^41,42^ This separation is favored by the high electrical conductivity inherent in MXene materials.^43^ In fact, the presence of a Schottky barrier at the interfacial region reduces the recombination of the photoinduced charge carriers, preventing the backward flow of electrons from the MXene to the TiO_2_.^41,42,44^

Even though there is a large number of studies focused on the experimental study of these systems, there is still a lack of knowledge regarding the interfacial structure and electronic properties of these heterostructures. Here, computational analyses make an important contribution, complementing experiments. For instance, recent previous studies have been dedicated to study the interfacial properties on several TiO_2_/MXenes composites,^45,46^ similar to the small TiO_2_ clusters formed at Ti-based MXenes upon exposure to air unveiled by STM in a recent study.^47^ In these theoretical studies, nanostructured (TiO_2_)_5_ and (TiO_2_)_10_ clusters have been supported on the bare Ti_2_C (0001) surface, showing a remarkable exothermic interaction coupled to a considerable charge transfer from the bare MXene surface towards TiO_2_ clusters.^45^ Notably, this strong interaction and charge transfer is found to be dependent on MXene metal composition.^46^ Although these studies shed light on important results, there is no analysis of the effect of the MXene termination on interface formation. As aforementioned, MXene surfaces are usually functionalized by some chemical groups, T_*x*_, and their role on the TiO_2_/MXene interface may play a key factor. Here, we focus on investigating the termination effect of Ti-based MXene on the structural and electronic properties of TiO_2_/MXene composites through first-principles calculations.

## 2 Models and Computational Details

**Figure 1:**
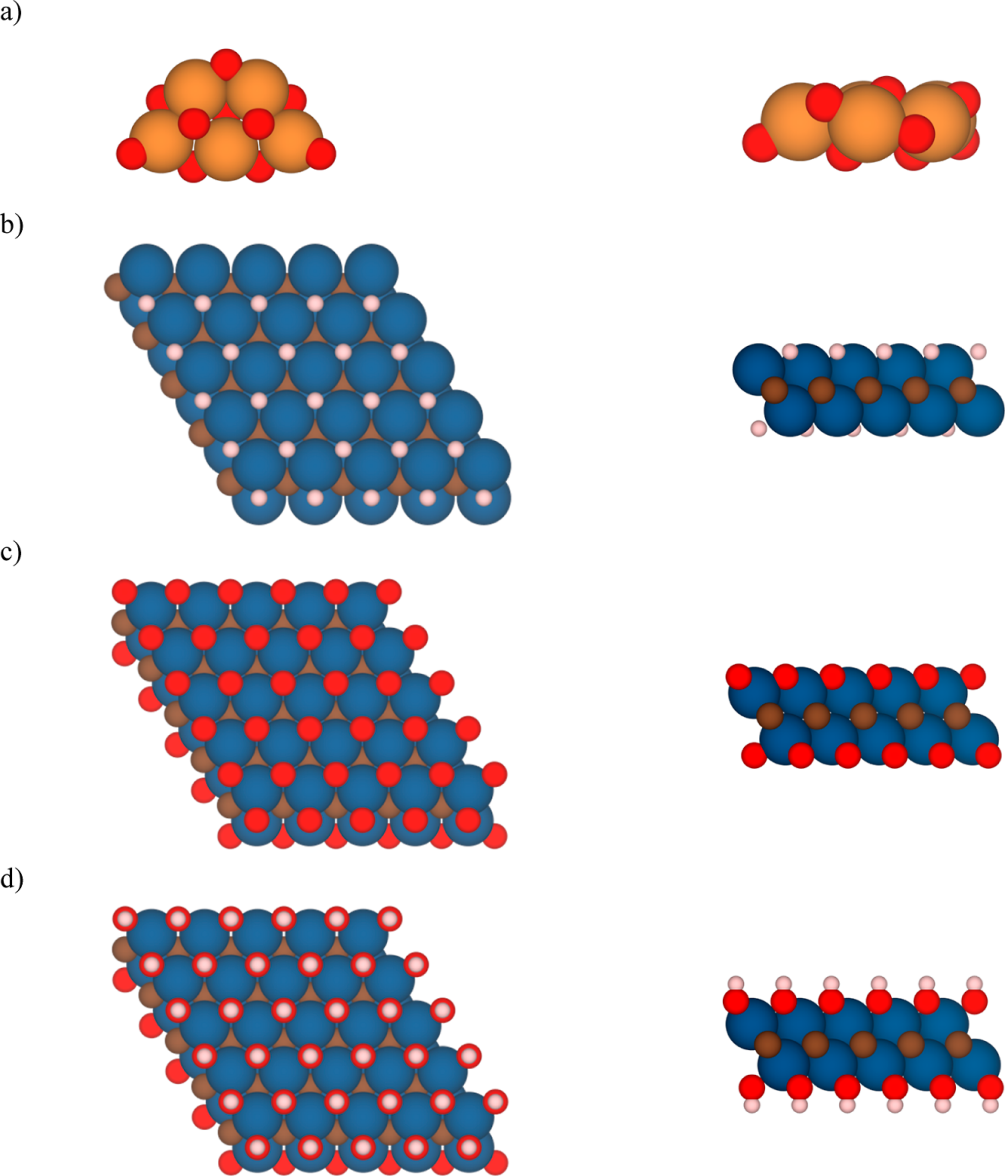
Top (left) and side (right) views of (a) planar (TiO_2_)_5_ cluster; the *p*(5 × 5) supercell model of the (0001) (b) Ti_2_CH_2_, (c) Ti_2_CO_2_, and (d) Ti_2_C(OH)_2_ MXene structures with a CABCA stacking. Orange and red spheres represent TiO_2_ Ti and O atoms, respectively, while blue, brown, and pale pink spheres represent the MXene Ti, C, and H atoms from Ti_2_CT_*x*_, respectively.

To study the role of MXene functionalization on the structure and electronic properties of TiO_2_/Ti-based MXenes composites, the extended Ti_2_CT_*x*_ (0001) surfaces with T_*x*_ = H, O, and OH are considered as support for the (TiO_2_)_5_ cluster. These functionalized surfaces have been modeled adsorbing H, O, and OH groups on the most favorable adsorption site of the ABC-stacked Ti_2_C(0001) surface, the hollow metal site, according to previous studies, leading to a resulting CABCA stacking for the functionalized surfaces.^48,49^ The gas-phase titania cluster atomic structure was obtained by global optimization methods, exhibiting the lowest energy in the potential energy surface (PES).^50,51^ Even though the size and morphology do not have a strong influence on the bonding nature of TiO_2_/MXene composites,^45^ we selected the (TiO_2_)_5_ cluster to support on MXene surfaces due to two reasons, (*i*) its planar initial geometry that could easily match the hexagonal MXene surfaces, leading to a high number of contacts, as we recently stablished in our previous study, and (*ii*) this facilitates the comparison with previous studies. Altogether, both surfaces and cluster models (see Figure 1) are representative cases to understand the chemical bond of TiO_2_/Ti-based MXene composites similar to the experimental systems observed by White *et al*.^47^ In order to find appropriate adsorption sites on the Ti_2_CT_*x*_ surfaces to anchor the titania cluster, scan of the PES has been carried out by generating different conformations via the rotation of the (TiO_2_)_5_ cluster over the Ti_2_CT_*x*_ (0001) MXene surfaces by means of a homemade python-based program.^52^ Various conformations were systematically generated by rotating the titania cluster with a step of 10°; this covers a total range of 140°. This systematic approach ensures a comprehensive study of PES, accounting for the inherent hexagonal symmetry of MXene. For further modeling details, we direct the readers to the literature.^45^ To accurately study the (TiO_2_)_5_/Ti_2_CT_*x*_ composites, a *p*(5 × 5) Ti_2_CT_*x*_ MXene supercell was chosen. This supercell is sufficiently large to minimize lateral interactions between the periodically repeated titania clusters as suitably selected in previous works.^45,46^

All calculations are carried out in the framework of the density functional theory (DFT) as implemented in Vienna *Ab initio* Simulation Package (VASP)^53,54^ using the well-known Perdew–Burke–Ernzerhof (PBE) exchange–correlation functional,^55^ within the generalized gradient approximation (GGA). The PBE functional is usually used in material science since it provides a good description of the lattice parameters and surface properties^56−58^ of functionalized MXenes, which are closed-shell metallic systems.^59,60^ In addition, the use of the PBE will allow us to compare the present results with previous studies.^45,46^ Furthermore, the dispersive (van der Waals) interactions between the (TiO_2_)_5_ cluster and Ti_2_CT_*x*_ MXene surfaces are accounted for by means of the zero-damping D3 method developed by Grimme.^61^ Kohn–Sham equations are numerically solved using a plane wave basis set to depict the valence electron density, with a kinetic energy cutoff of 415 eV. The interaction between the core and valence electron densities is handled using the projector augmented wave method,^62^ as implemented by Kresse and Joubert.^63^ All calculations have been performed with the non-spin-polarized restriction of the electronic density, since the functionalization of the (0001) Ti_2_C surface induces the fading out of the inherent magnetism found in Ti_2_C.^59,60^ A 15 Å vacuum space along the normal direction of the Ti_2_CT_*x*_(0001) surfaces was included since the (TiO_2_)_5_ cluster weight is around 2 Å, which leads to a vacuum thickness upon adsorption around 10 Å, large enough to avoid interactions between slab model replicas. Moreover, the numerical integrations in the reciprocal space are carried out at the Γ *k*-point only. Atomic relaxations were deemed to be converged when the forces acting on each nucleus fell below 0.01 eV/Å, and an energetic threshold criterion of 10^–6^ eV was set for electronic minimization.

First, the thermodynamic stability of (TiO_2_)_5_/Ti_2_CT_*x*_ (T_*x*_ = H, O, and OH) formation is studied based on the analysis of the adsorption energy, *E*_ads_. This quantity provides a measure of the adsorption process exothermicity (or endothermicity), and is defined as
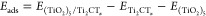
1where , , and  stand for the total energies of the (TiO_2_)_5_/Ti_2_CT_*x*_ composite, the Ti_2_CT_*x*_ (T_*x*_ = H, O, and OH) MXene, and the gas phase (TiO_2_)_5_ cluster, respectively. Following this *E*_ads_ definition, the adsorption process is considered thermodynamically favorable (*i.e.*, exothermic) when *E*_ads_ < 0. In addition, the composite formation is usually accompanied by deformations of each attachment component, X, in order to maximize the interaction, with deformation energy, *E*^def^, defined as
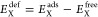
2where ads and free refer to adsorbed and pristine/vacuum geometries, respectively. However, this deformation energy cost is normally compensated by the interaction strength, also known as adhesion energy, *E*_adh_, defined as
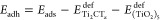
3

This adhesion energy quantifies the interaction strength between the already deformed fragments; thus, a more negative value indicates a stronger attachment.

Afterwards, the chemical interaction is studied by means of the topological analysis of the electron density, based on the Bader charges net change upon adsorption. This population analysis employs the Bader approach of Atoms-in-Molecules (AIM) using the VASP-linked code built by Henkelman *et al*.^64^ This analysis has been performed at each structure in its gas phase and composite geometry to understand the effect of the adsorption process on the bonding nature, as it quantifies the possible charge transfer between the adsorption building blocks. Furthermore, the Bader charge picture is complemented with a more visual and qualitative analysis through the Charge Density Difference (CDD) and the Plane-Averaged CCD (PA-CCD) analyses, where both are based on density differences, Δ*ρ*, defined as
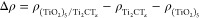
4where Δ*ρ* corresponds to CDD, , , and  represent the charge density of (TiO_2_)_5_/Ti_2_CT_*x*_, Ti_2_CT_*x*_, and (TiO_2_)_5_ systems, respectively, at the adsorbed composite geometry. The CDD and PA-CDD calculations have been handled with the command-line VASPKIT program.^65^

Finally, to achieve a more accurate depiction of the electronic structure and address the limitations of GGA-derived functionals, such as the well-known underestimation of the band gap,^66^ we carried out additional electronic structure analysis using single-point calculations with the hybrid Heyd–Scuseria–Ernzerhof (HSE06) exchange–correlation functional on the relaxed PBE geometries. This hybrid functional includes a 25% of Fock exchange with a screening parameter, ω, of 0.2 Å^–1^ in order to improve computational efficiency for closed-shell and metallic systems^67^ like functionalized MXenes are known to be.^59,60^ This hybrid functional allows achieving more reliable electronic structure results, which is also useful when analyzing the Density Of States (DOS) of each composite and its individual building blocks. At this point, we point out that the hybrid HSE06 density functional is not the best choice to describe the electronic structure of TiO_2_ phases because it underestimates their band gap.^68^ Such underestimation may be solved through self-interaction-corrected methods, such as the atomic-orbital-based self-interaction correction (ASIC) or Wannier–Fermi–Löwdin schemes.^69,70^ In fact, other hybrid density functionals such as PBE*x* density functional, a modified hybrid PBE0 with a 12.5% of non-local Fock exchange, leads to an accurate description of TiO_2_ electronic and structural properties.^71^ However, since we are dealing with a system composed by two materials of different nature, a TiO_2_ semiconductor anchored to MXenes, we have chosen the HSE06 functional as a good balance between accuracy and computational cost. In addition, we also want to point out that GW calculations could be the appropriate method as reported recently in MXenes.^72^ However, the derived computational cost of such calculations is unaffordable for the systems investigated in the present study.

## 3 Results and Discussion

### 3.1 Thermodynamic Stability of (TiO_2_)_5_/Ti_2_CT_*x*_ (T_*x*_ = H, O, and OH) Composites

We start by discussing the MXene functionalization role on the aforementioned energetic quantities when the (TiO_2_)_5_ cluster is adsorbed over the (0001) Ti_2_CT_*x*_ (T_*x*_ = H, O, and OH) surface. First, each composite was constructed by positioning the cluster above each surface and rotating it as described in the previous section, followed by a full, unconstrained relaxation step. Afterwards, the most stable configuration—the one with the largest *E*_ads_ in absolute value—is selected from among the various configurations with different initial rotating angles for each system in order to analyze the energetic descriptors of each functionalized surface. Table 1 gathers the *E*_ads_, *E*_adh_, , and  for the most stable configurations of (TiO_2_)_5_/Ti_2_CT_*x*_ (T_*x*_ = H, O, and OH) composites; see Tables S1–S3 in the Supporting Information (SI) for further data.

**Table 1:** *E*_ads_ and *E*_adh_, given in eV, for the Most Favorable Configuration of (TiO_2_)_5_/Ti_2_CT_*x*_ (T_*x*_ = H, O, and OH) Composites. The (TiO_2_)_5_ Cluster and the Ti_2_CT_*x*_ Surface Deformation upon Adsorption Are Included As  and , respectively, Also Given in eV. The Change on The (TiO_2_)_5_ Bader Charges upon Adsorption, Δ*Q*, Are Given in e.

Ti_2_CT_*x*_	*E*_ads_	*E*_adh_	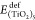	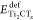	Δ*Q*
Ti_2_CH_2_	–6.17	–11.16	2.93	2.06	–1.45
Ti_2_CO_2_	–2.76	–5.22	0.99	1.47	0.15
Ti_2_C(OH)_2_	–6.18	–12.17	4.99	1.01	–2.30

*E*_ads_ from Table 1 shows negative values of −6.17, −2.76, and −6.18 eV for the most stable configurations of (TiO_2_)_5_/Ti_2_CH_2_, (TiO_2_)_5_/Ti_2_CO_2_, and (TiO_2_)_5_/Ti_2_C(OH)_2_ composites, respectively, leading to an exothermic interaction between the titania cluster and MXene surfaces. Clearly, MXene terminations promote different adsorption energies, providing the smallest one for the case of the (TiO_2_)_5_/Ti_2_CO_2_ composite, whereas with H and OH terminations, the *E*_ads_ are nearly the same, suggesting a stable adsorption of the (TiO_2_)_5_ cluster over the Ti_2_CH_2_ and Ti_2_C(OH)_2_ surfaces and less stable over the Ti_2_CO_2_. This could be rooted in the fact that the −O terminated MXene exposing an oxygen atomic layer on its surface can lead to repulsive interactions with the O atoms from the titania cluster, leading also to a smaller *E*_ads_.

In addition, the influence of the dispersion term can be studied by comparing the PBE-D3 values versus the PBE ones. In that way, the contribution of the dispersion term to the *E*_ads_ is −2.54, −1.70, and −1.60 eV for (TiO_2_)_5_/Ti_2_CH_2_, (TiO_2_)_5_/Ti_2_CO_2_, and (TiO_2_)_5_/Ti_2_C(OH)_2_, respectively. In that sense, these values indicate that there is relevant van der Waals interactions between the titania cluster and MXene surfaces. These interactions are similar when the MXene surface is functionalized with −O and −OH, while it is stronger when −H termination covers the Ti_2_C(0001) surface, the last one being the composite with the largest dispersion contributions. As it will be discussed in the next section, the (TiO_2_)_5_/Ti_2_CH_2_ composite possesses a larger number of contacts between the (TiO_2_)_5_ cluster and Ti_2_CH_2_ surface, promoting a larger contribution of the dispersion term of the *E*_ads_ and *E*_adh_. Overall, these *E*_ads_ are not so exothermic when comparing with the interaction of (TiO_2_)_5_ cluster with the bare Ti_2_C(0001) surface (*ca*. −16.76 eV).^45^

Next, we focus on *E*_adh_, which is also listed in Table 1. This energy parameter mirrors the trend observed in the *E*_ads_, and all MXene terminations show significantly negative values, indicating a relatively strong *E*_adh_ with high stability and bonding strength. Among the terminations, the O-terminated MXene exhibits the lowest overall interaction strength (−5.22 eV), reflecting a weaker general bonding strength with the (TiO_2_)_5_ cluster. As mentioned above, this could also be rooted in the repulsion of O–O atoms from TiO_2_ and Ti_2_CO_2_. Note the high difference between *E*_adh_ and *E*_ads_ values, implying that the deformation is a key factor in the adsorption process. In contrast, −H and −OH terminations display higher *E*_adh_ values due to a higher interaction strength. In contrast to *E*_ads_, the influence of MXene termination arises, providing slight differences in *E*_adh_ that lead to a slightly weaker interaction for the −H termination (−11.16 eV), 1 eV smaller than that of the −OH termination (−12.17 eV). Since the (TiO_2_)_5_/Ti_2_CH_2_ composite presents a larger number of contacts, in comparison to (TiO_2_)_5_/Ti_2_CO_2_ and (TiO_2_)_5_/Ti_2_C(OH)_2_ ones, normalizing the *E*_adh_ by the number of interactions leads to values of −1.02, −1.74, and −1.74 eV/number of interactions for (TiO_2_)_5_/Ti_2_CH_2_, (TiO_2_)_5_/Ti_2_CO_2_, and (TiO_2_)_5_/Ti_2_C(OH)_2_, respectively, which implies that the strength of each bond in the (TiO_2_)_5_/Ti_2_CH_2_ composite is smaller in comparison to the other two composites, presenting similar values.

**Figure 2:**
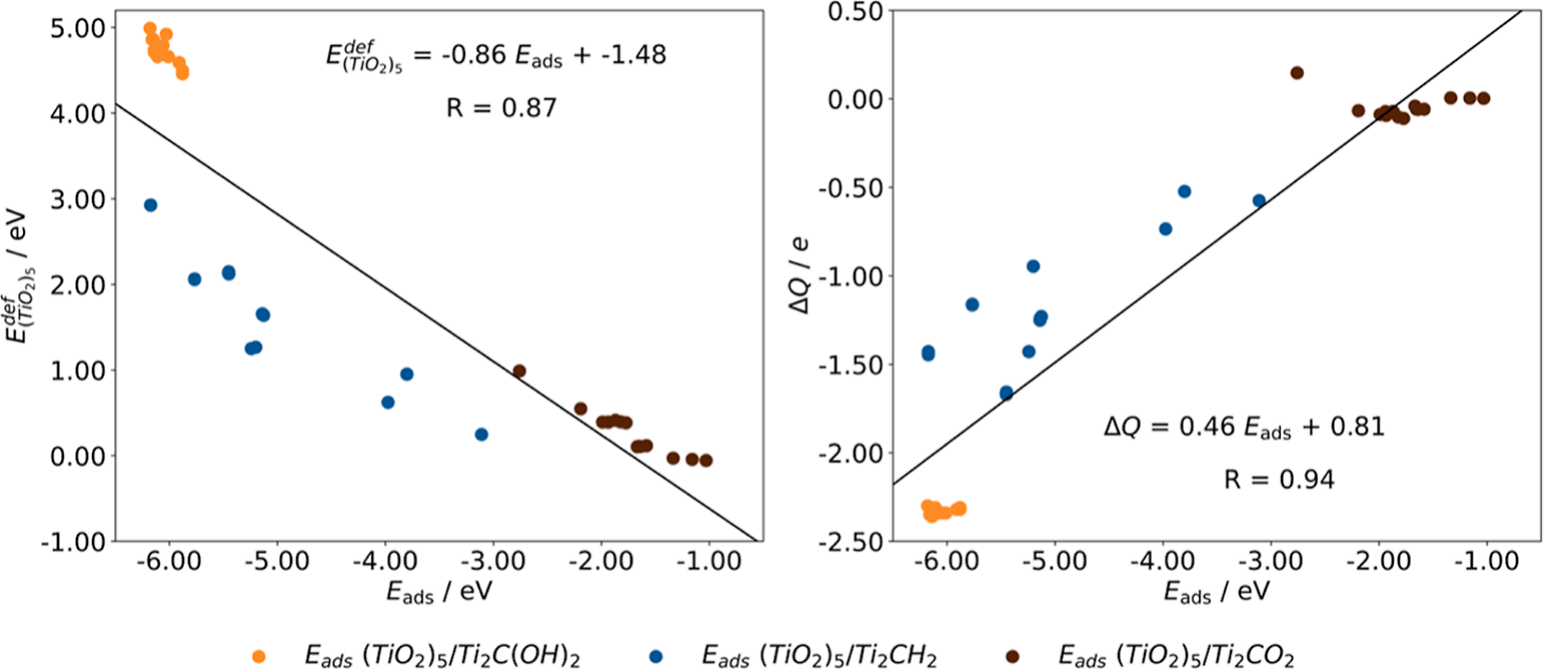
Linear adjustment, black lines, between  and Δ*Qvs*. *E*_ads_ of (TiO_2_)_5_/Ti_2_CT_*x*_ composites, color-coded.

The interaction between these systems is usually accompanied by a structural deformation of each part. All of these structural changes can be quantified by the deformation energies, as listed in Table 1. These deformation energies of the Ti_2_CT_*x*_ surfaces show notable differences. For instance, Ti_2_CH_2_ has a deformation energy of 2.06 eV, indicating moderate structural alterations, while Ti_2_CO_2_ has an even lower deformation energy of 1.47 eV, suggesting fewer structural alterations. Finally, Ti_2_C(OH)_2_ displays the smallest deformation energy of 1.01 eV, which corresponds to the least structural modification among the investigated cases. For the (TiO_2_)_5_ cluster, its deformation energy also varies across the composites. For instance, on Ti_2_CH_2_ surface (TiO_2_)_5_ exposes a high cluster deformation (2.93 eV), suggesting significant structural changes in the (TiO_2_)_5_ upon adsorption. Over the Ti_2_C(OH)_2_ surface, (TiO_2_)_5_ suffers even more deformation with an energy change of 4.99 eV. Finally, on the Ti_2_CO_2_ surface, the smallest deformation energy is achieved with an energy of 0.99 eV, implying that the structural disruption in the cluster upon adsorption has not a large energetic impact on the composite formation. In addition, the cluster that presents the smaller deformation upon adsorption should be the one closer to the global minima of the PES, *i.e.*, the initial geometry. The results show that the (TiO_2_)_5_ anchored to the Ti_2_CO_2_ (0001) surface is the most stable adsorbed cluster, with the smallest deformation energy of 0.99 eV. This could be rooted to the fact that for the other composites, some O have changed plains to maximize the number of contacts, increasing the repulsion between O atoms within the cluster and, thus, turning the (TiO_2_)_5_ anchored to the Ti_2_CO_2_(0001) surface the most stable cluster upon adsorption. To generate a more general picture of these energetic quantities, an overall trend can be obtained by facing  vs *E*_ads_ of (TiO_2_)_5_/Ti_2_CT_*x*_ as shown in Figure 2. There, the higher the , the stronger the *E*_ads_.

**Figure 3:**
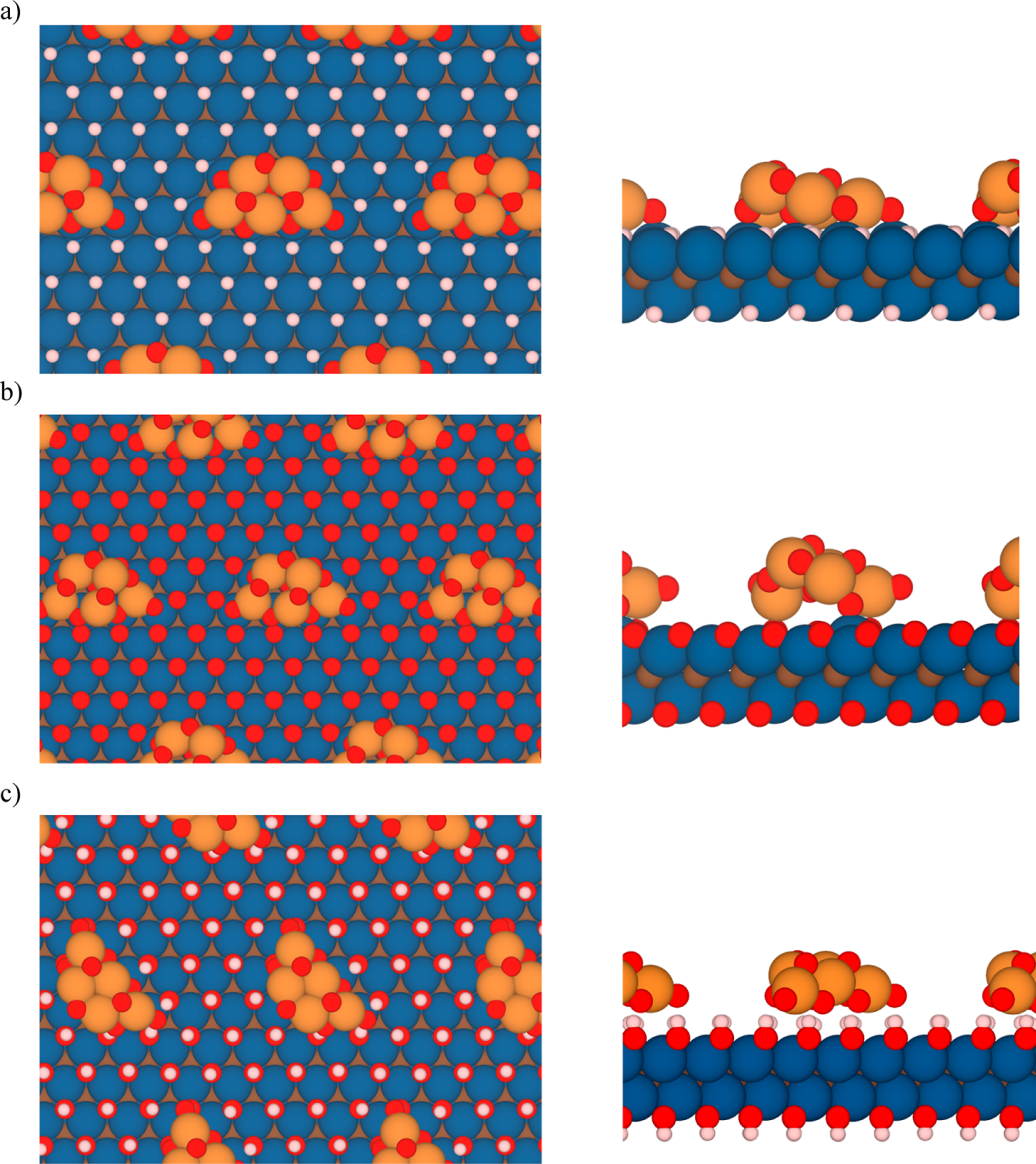
Top (left) and side (right) views of the most stable configuration of (a) (TiO_2_)_5_/Ti_2_CH_2_, (b) (TiO_2_)_5_/Ti_2_CO_2_, and (c) (TiO_2_)_5_/Ti_2_C(OH)_2_ composites. Color scheme as in Figure 1.

The fully relaxed geometries of the most energetically favorable configurations of each composite before and after adsorption are depicted in Figures 1 and 3, respectively. There, clear deformations of (TiO_2_)_5_ clusters upon adsorption over the (0001) Ti_2_CT_*x*_ surfaces are shown, in full concordance with deformation energy values from Table 1, with the driving force being the maximization of interactions. For the (TiO_2_)_5_/Ti_2_CH_2_ case, seven of the ten (TiO_2_)_5_ O atoms occupy a high-symmetry site on the Ti_2_CH_2_ surface, actually on top of the MXene Ti atoms, since hollow metal sites are already occupied by H atoms, even if such sites would be the anchoring points of O atoms on pristine Ti_2_C MXene.^73^ In fact, there are structural changes of the TiO_2_ cluster to have more of the O atoms in contact with the −H terminated MXene surface (Figure 3a). However, for the (TiO_2_)_5_/Ti_2_CO_2_ composite, the number of contacts is smaller than that of the (TiO_2_)_5_/Ti_2_CH_2_, with two titania Ti atoms interacting with the MXene surface O atoms and one TiO_2_ O atom on top of a MXene Ti atom (Figure 3b). Indeed, this interaction leads to the titania cluster losing its gas-phase planar geometry. These qualitative structural aspects are in line with the aforementioned analysis of *E*_ads_ and *E*_adh_. Finally, for the (TiO_2_)_5_/Ti_2_C(OH)_2_ composite, the titania O atoms and −OH MXene surface terminations align together, and as for the (TiO_2_)_5_/Ti_2_CH_2_, some titania O atoms suffer structural changes to enter in contact with the Ti_2_C(OH)_2_ (0001) surface (see Figure 3c).

### 3.2 Interface Polarization

The charge density changes upon (TiO_2_)_5_ cluster adsorption over Ti_2_CT_*x*_ surfaces have been investigated by means of Bader charges, CDD, and PA-CDD. First, as far as Bader charges are concerned, the topological and population analysis has been performed on every structure before and after the composite formation, which allows one to quantify the charge transfer and its transferring direction. Table 1 compiles the change in total net charges of the (TiO_2_)_5_ cluster upon adsorption, Δ*Q*, for each composite at its most stable configuration, while the data for the other configurations are gathered in Tables S1–S3 of the SI. Since the isolated titania cluster is neutral, a negative sign of Δ*Q* indicates an electron accumulation on the supported (TiO_2_)_5_ cluster upon adsorption, as a result from an electron transfer from the Ti_2_CT_*x*_ surfaces towards (TiO_2_)_5_, whereas a positive sign indicates an electron transfer from (TiO_2_)_5_ towards the Ti_2_CT_*x*_ MXenes. Table 1 values show a significant electron accumulation on the (TiO_2_)_5_ cluster when the cluster is adsorbed onto the Ti_2_CH_2_ and Ti_2_C(OH)_2_ surfaces, of −1.45 and −2.30 *e*, respectively. On the other hand, when the Ti_2_C surface is functionalized with oxygens, there is a nearly negligible electron depletion of 0.15 *e* on the (TiO_2_)_5_ cluster, in line with the relatively small interaction strength and also indicative of a small electronic rearrangement. It is worth highlighting another linear trend can be extrated studying the interface polarization. the larger the charge transfer, Δ*Q*, the stronger the *E*_ads_, as is shown in Figure 2

**Figure 4:**
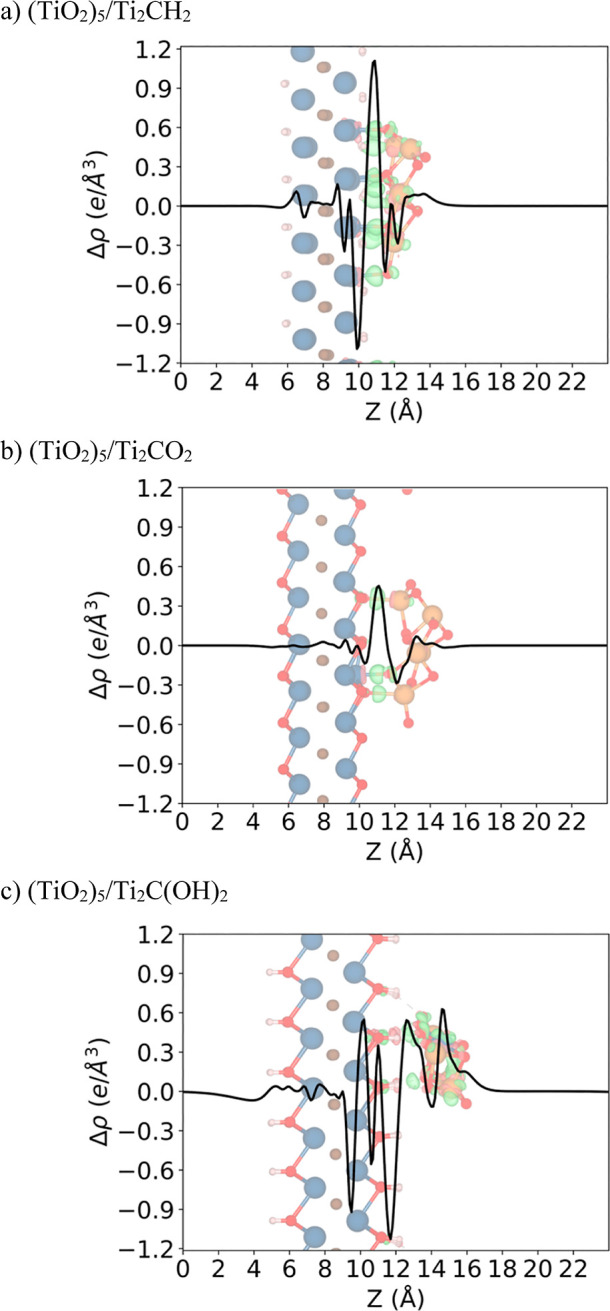
PA-CDDs (Δ*ρ* in e/Å^3^) along the normal direction to the MXene surface for the most stable configuration of (a) (TiO_2_)_5_/Ti_2_CH_2_, (b) (TiO_2_)_5_/Ti_2_CO_2_, and (TiO_2_)_5_/Ti_2_C(OH)_2_ composites. CDDs with an isosurface value of 0.01 *e*/Å^3^ are depicted inset, where green and red isosurfaces correspond to charge accumulation and depletion, respectively. Color scheme as in Figure 1.

When inspecting CDD and the PA-CDD, the last along the normal direction of Ti_2_CT_*x*_ surfaces (see Figure 4), the PA-CDD and inset CDD of (TiO_2_)_5_/Ti_2_CH_2_ (see Figure 4a) show a strongly localized charge rearrangement at the interface, mainly due to the bond formation, where the electron density is mainly depleted from the MXene substrate, while it is accumulated in the (TiO_2_)_5_ cluster, in agreement with Bader charges interpretation. Indeed, from the CDD analysis inset in Figure 4a, there is a charge accumulation at the interface region indicating four bond formations between the H atoms from Ti_2_CH_2_ and Ti atoms from the (TiO_2_)_5_ cluster besides the seven ones between the Ti atoms from Ti_2_CH_2_ and the O atoms from the (TiO_2_)_5_ cluster. Next, for (TiO_2_)_5_/Ti_2_CO_2_ (see Figure 4b), the PA-CDD and CDD show a weak charge localization at the interface, where the electrons involved in the bond formation mainly come from the (TiO_2_)_5_ cluster and from bonds between one Ti atom from Ti_2_CO_2_ and the O atom from the (TiO_2_)_5_ cluster along with two Ti_2_CO_2_ O atoms and (TiO_2_)_5_ Ti atoms. Finally, for (TiO_2_)_5_/Ti_2_C(OH)_2_ (see Figure 4c), the charge rearrangement is not localized at the interface; nevertheless, there is an electron depletion at the Ti_2_C(OH)_2_ surface and an electron accumulation at the (TiO_2_)_5_ cluster. Note that in this case, the CDD plot shows bond formations between the Ti Ti_2_C(OH)_2_ H atoms and the O (TiO_2_)_5_ cluster atoms. This charge rearrangement is also consistent with the information obtained through the Bader charges analysis. Furthermore, for the cases where there is an electron transfer from the Ti_2_CT_*x*_ MXenes towards (TiO_2_)_5_ clusters, this charge rearrangement could generate a build-in electric field near the interface, which may be advantageous in photocatalytic applications by acting as a possible charge carriers’ separator.^74^

### 3.3 Electronic Structure Analysis

**Figure 5:**
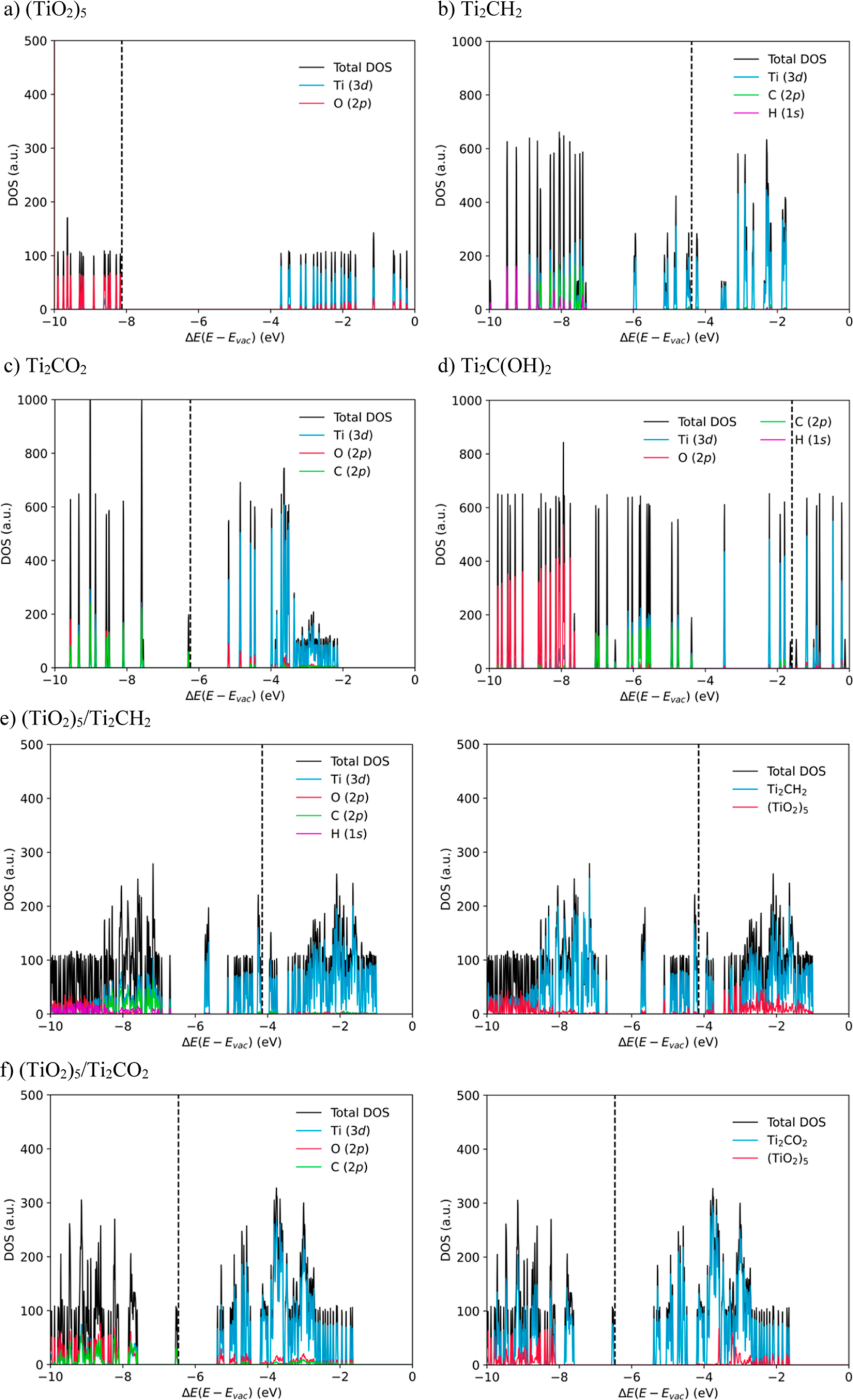

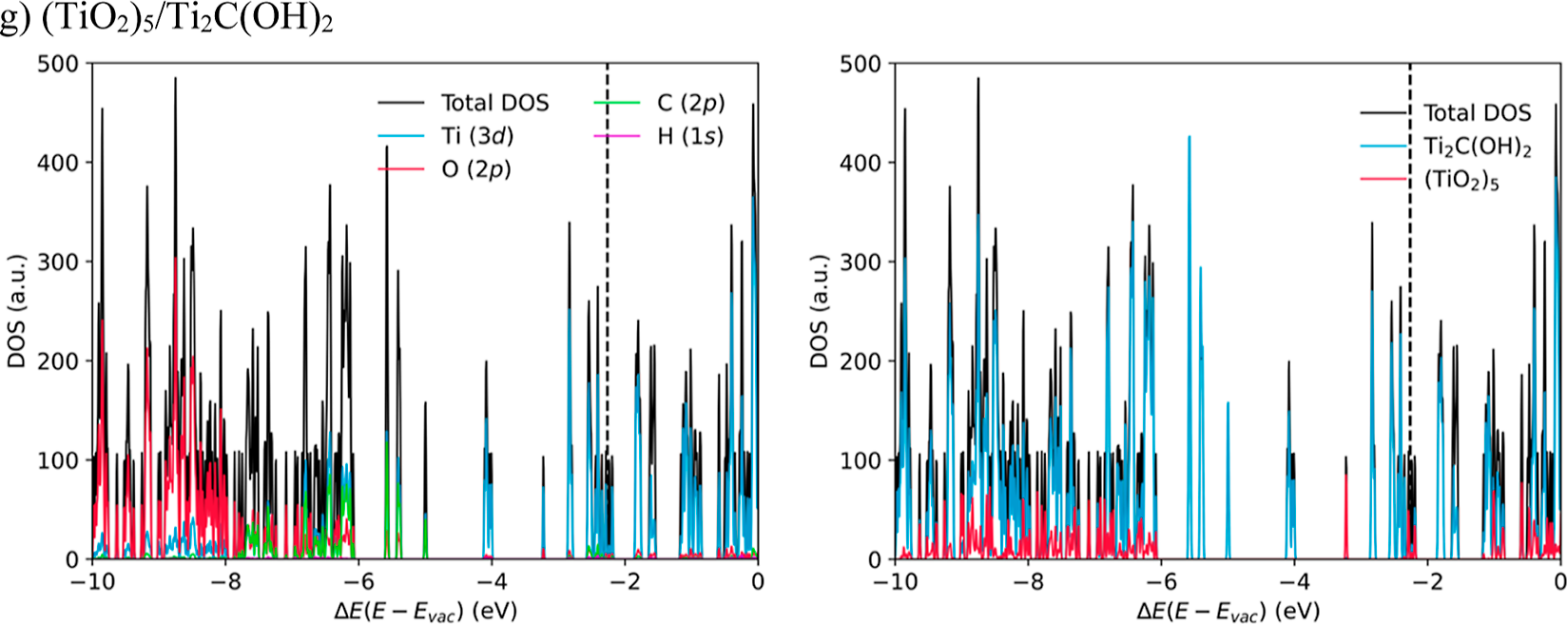
PDOS decomposed per atomic orbital contributions, in a.u., for (a) (TiO_2_)_5_, (b) Ti_2_CH_2_, (c) Ti_2_CO_2_, and (d) Ti_2_C(OH)_2_. PDOS decomposed per atomic orbital contributions (left) and PDOS per composite part (right), in a.u., for (e) (TiO_2_)_5_/Ti_2_CH_2_, (f) (TiO_2_)_5_/Ti_2_CO_2_, and (g) (TiO_2_)_5_/Ti_2_C(OH)_2_. All PDOS are single-point HSE06 estimates on PBE geometries. The energies, *E* in eV, are referenced to the vacuum level, *E*_vac_, while the Fermi level is marked with a dashed line.

To overcome the well-known drawbacks of GGA functionals in the depiction of the electronic structure of insulating and semiconducting materials,^66^ results in this section are obtained employing the hybrid HSE06 functional at the PBE structures. The Projected DOS (PDOS) per atomic orbital of each interaction part (titania cluster or MXene substrate) is gathered in Figure 5. These plots, together with the PDOS shown in Figure 5 allow us to reveal the influence of the composite formation on the electronic structure of each component.

Focusing on the electronic structure of each part before the composite formation (see Figure 5a–d), the (TiO_2_)_5_ cluster shows a relative wide band gap of 4.5 eV, higher than the that of the anatase bulk structure, 3.2 eV,^75^ due to the quantum confinement, and also somehow the treatment of the non-local Fock exchange of the DFT functional. The valence states mainly belong to the O(2*p*), and the virtual ones, to Ti(3*d*). These available and empty Ti(3*d*) states could be filled in the charge transfer process directed towards the TiO_2_ Ti atoms from the Ti_2_CT_*x*_ surfaces. In contrast, the DOS of the Ti_2_CH_2_ and Ti_2_C(OH)_2_ MXenes shows an almost gapless electronic structure (0.1 eV), in line with previous estimates,^28^ indicating a general metallic character with broad bands dominated by Ti(3*d*) contribution near the Fermi level as in the case of the bare Ti_2_C surface.^76^ However, this general metallic character is not preserved when the Ti_2_C surface is functionalized with O atoms, broadening the band gap up to 1.1 eV, also in line with previous estimates of 1.64 eV using the PBE0 hybrid functional.^77^ It is worth highlighting the fact that different MXene functionalizations lead to different work functions for each surface, *i.e*., 4.4, 6.5, and 1.6 eV for Ti_2_CH_2_, Ti_2_CO_2_, Ti_2_C(OH)_2_, respectively. These differences in the work functions promote different Fermi energy levels shown in Figure 5b–d.

Upon (TiO_2_)_5_/Ti_2_CH_2_ and (TiO_2_)_5_/Ti_2_C(OH)_2_ composite formation (*cf*. Figure 5e–g), the general metallic character remains as the (TiO_2_)_5_ cluster is adsorbed, with the O(2*p*) states lying way below the Fermi energy level, while the Ti(3*d*) states continue to surround it. In fact, the main contribution to the conduction band is still dominated by the Ti(3*d*) states for all cases. On the other hand, when the (TiO_2_)_5_ cluster is adsorbed on the Ti_2_CO_2_ surface, the 1.1 eV MXene band gap is preserved, and the valence band is more filled with O(2*p*) states coming from the titania cluster. Note that, for all cases, the TiO_2_ valence band presents a strong mixing of orbitals, similarly to previous studies involving the (TiO_2_)_5_/Ti_2_C composite.^45^ By projecting the DOS onto each part of the composite, it becomes evident that MXene states fill the TiO_2_ band gap and the primary contribution to the bands near the Fermi level are originated by the Ti_2_CH_2_ and Ti_2_C(OH)_2_ components (as shown in Figure 5e,g). However, a Ti(3*d*) band from the titania cluster is also visible and near the Fermi level, suggesting that the electronic structures of the titania clusters and both MXenes are highly integrated. This merging results in the composite system exhibiting a general metallic character. Nevertheless, for the (TiO_2_)_5_/Ti_2_CO_2_ composite (*cf*. Figure 5f), part of the valence and conduction bands of Ti_2_CO_2_ are placed inside the TiO_2_ band gap, similar to the band positions in a Type-I heterojunction.

## 4 Conclusions

The functionalization effect of the Ti_2_CT_*x*_ MXene on the structure, bonding nature, and electronic properties of the (TiO_2_)_5_ cluster supported upon (T_*x*_ = H, O, and OH) (0001) surfaces is described here based on DFT calculations. The interaction with the (TiO_2_)_5_ cluster is exothermic regardless of the surface termination, but the functionalization effect arises showing different adsorption energies of −6.17, −2.76, and −6.18 eV for (TiO_2_)_5_/Ti_2_CH_2_, (TiO_2_)_5_/Ti_2_CO_2_, and (TiO_2_)_5_/Ti_2_C(OH)_2_ composites, respectively. Results show that the functionalization also drives the bond strength between the TiO_2_ cluster and the MXene surface, leading to a higher number of contacts and weaker bonds when the MXene is terminated with H atoms in comparison with −O and −OH groups. Additionally, the interaction between these systems often leads to structural deformation of each part, analyzed in terms of a cost–benefit relationship based on deformation and adhesion energies. Moreover, the found linear trend suggests that *E*_ads_ is directly proportional to  on (TiO_2_)_5_/Ti_2_CT_*x*_ composites.

The nature of the interaction when adsorbing the (TiO_2_)_5_ cluster over the Ti_2_CT_*x*_ surfaces has been investigated by means of Bader charges analysis. It reveals a charge transfer from Ti_2_CH_2_ and Ti_2_C(OH)_2_ surfaces towards the (TiO_2_)_5_ cluster of −1.45 and −2.30 *e*, respectively. However, when the titania cluster is adsorbed on Ti_2_CO_2_, there is almost negligible electron accumulation in the MXene surfaces and a depletion in the titania cluster of 0.15 *e*. In addition, Δ*Q* is also directly proportional to *E*_ads_ of (TiO_2_)_5_/Ti_2_CT_*x*_ composites (see Figure 2). This population analysis of the charge density has been complemented by the study of the CDD and PA-CDD, showing a charge rearrangement localized at the interface when the titania cluster is supported on Ti_2_CH_2_, and a smaller one when is on the Ti_2_CO_2_ surface, whereas the charge rearrangement is delocalized when the same cluster is supported on the Ti_2_C(OH)_2_ surface.

Finally, the electronic structure of each composite was studied by employing the hybrid HSE06 density functional. The DOS analysis of the isolated parts shows a band gap of 4.5 and 1.1 eV for the (TiO_2_)_5_ cluster and Ti_2_CO_2_ MXene, respectively, while for the other two MXene surfaces, Ti_2_CH_2_ and Ti_2_C(OH)_2_, an almost gapless metallic electronic structure is found. The isolated titania cluster band gap disappears upon the composite formation, leading to two essentially metallic systems when it is supported on the Ti_2_CH_2_ and Ti_2_C(OH)_2_ surfaces, with titania Ti(3*d*) states hybridized with the MXene Ti(3*d*) band giving rise to a titania band near the Fermi level. However, the electronic structure of (TiO_2_)_5_/Ti_2_CO_2_ shows a semiconducting–semiconducting interface, with the unchanged band edges in positions common in a Type-I heterojunction.

All in all, for (TiO_2_)_5_/Ti_2_CH_2_ and (TiO_2_)_5_/Ti_2_CO_2_ composites, all analyses lead to the conclusion that the MXene functionalization has a strong effect on the TiO_2_/MXene interaction, this being promoted when the MXene surface is functionalized with −H and −OH groups, where the adsorption process is accompanied by a significant charge transfer.

## Supporting Information Available

Full analysis of adsorption and adhesion energies at different rotated (TiO_2_)_5_ cluster angles for (TiO_2_)_5_/Ti_2_CH_2_, (TiO_2_)_5_/Ti_2_CO_2_, and (TiO_2_)_5_/Ti_2_C(OH)_2_ composites; and deformation energies and the (TiO_2_)_5_ Bader charges (PDF)
