# Supplementary material for: Effect of the Ti2CTx (Tx = O, OH, and H) Functionalization on the Formation of (TiO2)5/Ti2CTx Composites
Source: J Phys Chem C Nanomater Interfaces. 2024 Dec 19;129(1):826–36. doi: 10.1021/acs.jpcc.4c06909 (PMC11726659; doi:10.1021/acs.jpcc.4c06909)
Supplement: Supplementary file 1 — jp4c06909_si_001.pdf [file jp4c06909_si_001.pdf]

Effect of the Ti_2CT_x (T_x = O, OH, and H) Functionalization on the Formation of $(TiO_2)_5/Ti_2CT_x$ Composites

Néstor García-Romeral, Ángel Morales-García,* Francesc Viñes

Departament de Ciència de Materials i Química Física & Institut de Química Teòrica i Computacional (IQTCUB), Universitat de Barcelona, c/ Martí i Franquès 1-11, 08028 Barcelona, Spain.

*e-mail: angel.morales@ub.edu

Table S1. Adsorption, E_{ads}, and adhesion, E_{adh}, energies, given in eV of $(TiO_2)_5/Ti_2CH_2$ composites at different rotated $(TiO_2)_5$ cluster angle, α. The deformation of the $(TiO_2)_5$ cluster and the one of the Ti_2CH_2 surface upon adsorption are included as $E_{(TiO_2)_5}^{def}$ and $E_{Ti_2CH_2}^{def}$, respectively, also given in eV. The $(TiO_2)_5$ Bader charges change upon adsorption, ΔQ, is also given, in *e*. The row in bold corresponds to the most stable configuration.

α	E_{ads}	E_{adh}	$E_{(TiO_2)_5}^{def}$	$E_{Ti_2CH_2}^{def}$	ΔQ
**0**	**-6.17**	**-11.16**	**2.93**	**2.06**	**-1.45**
10	-6.17	-11.17	2.93	2.07	-1.43
20	-5.24	-7.89	1.25	1.40	-1.43
30	-3.11	-3.93	0.25	0.57	-0.58
40	-5.45	-9.69	2.12	2.11	-1.66
50	-5.13	-8.30	1.64	1.54	-1.23
60	-5.14	-8.32	1.65	1.53	-1.24
70	-5.14	-8.33	1.66	1.53	-1.25
80	-5.45	-9.69	2.13	2.11	-1.66
90	-5.45	-9.72	2.15	2.12	-1.67
100	-3.80	-5.66	0.95	0.91	-0.52
110	-5.20	-7.93	1.27	1.47	-0.95
120	-5.77	-9.40	2.06	1.58	-1.16
130	-5.77	-9.41	2.06	1.59	-1.16
140	-3.98	-6.08	0.62	1.48	-0.73

Table S2. Adsorption, E_{ads}, and adhesion, E_{adh}, energies, given in eV of $(TiO_2)_5/Ti_2CO_2$ composites at different rotated $(TiO_2)_5$ cluster angle, α. The deformation of the $(TiO_2)_5$ cluster and the one of the Ti_2CO_2 surface upon adsorption are included as $E^{def}_{(TiO_2)_5}$ and $E^{def}_{Ti_2CO_2}$, respectively, also given in eV. The $(TiO_2)_5$ Bader charges change upon adsorption, ΔQ, is also given, in *e*. The row in bold corresponds to the most stable configuration.

α	E_{ads}	E_{adh}	$E^{def}_{(TiO_2)_5}$	$E^{def}_{Ti_2CO_2}$	ΔQ
0	-2.19	-2.98	0.55	0.24	-0.07
**10**	**-2.76**	**-5.22**	**0.99**	**1.47**	**0.15**
20	-1.64	-1.81	0.11	0.06	-0.06
30	-1.03	-0.87	-0.06	-0.11	0.00
40	-1.82	-2.39	0.40	0.17	-0.10
50	-1.87	-2.47	0.41	0.19	-0.07
60	-1.34	-1.24	-0.03	-0.07	0.01
70	-1.16	-1.00	-0.04	-0.11	0.00
80	-1.77	-2.33	0.38	0.17	-0.11
90	-1.94	-2.53	0.39	0.20	-0.07
100	-1.58	-1.76	0.12	0.06	-0.06
110	-1.65	-1.82	0.11	0.06	-0.06
120	-1.67	-1.84	0.10	0.07	-0.04
130	-1.93	-2.50	0.39	0.18	-0.09
140	-1.99	-2.57	0.39	0.19	-0.09

Table S3. Adsorption, E_{ads}, and adhesion, E_{adh}, energies, given in eV of $(TiO_2)_5/Ti_2C(OH)_2$ composites at different rotated $(TiO_2)_5$ cluster angle, α. The deformation of the $(TiO_2)_5$ cluster and the one of the $Ti_2C(OH)_2$ surface upon adsorption are included as $E_{(TiO_2)_5}^{def}$ and $E_{Ti_2C(OH)_2}^{def}$, respectively, also given in eV. The $(TiO_2)_5$ Bader charges change upon adsorption, ΔQ, is also given, in *e*. The row in bold corresponds to the most stable configuration.

α	E_{ads}	E_{adh}	$E_{(TiO_2)_5}^{def}$	$E_{Ti_2C(OH)_2}^{def}$	ΔQ
0	-6.01	-11.45	4.66	0.78	-2.34
10	-6.11	-11.55	4.66	0.78	-2.34
20	-5.88	-11.31	4.46	0.98	-2.31
30	-5.88	-11.36	4.50	0.98	-2.32
40	-6.06	-11.87	4.79	1.01	-2.34
50	-6.14	-11.67	4.72	0.80	-2.36
60	-6.14	-11.69	4.74	0.81	-2.32
70	-6.14	-11.66	4.71	0.80	-2.32
80	-5.91	-11.54	4.59	1.04	-2.32
90	-6.03	-12.06	4.92	1.11	-2.34
100	-6.15	-11.92	4.86	0.91	-2.35
110	-6.12	-11.59	4.69	0.79	-2.35
120	-6.11	-11.55	4.66	0.78	-2.31
130	-6.16	-11.92	4.86	0.90	-2.35
**140**	**-6.18**	**-12.17**	**4.99**	**1.01**	**-2.30**
